# Moral expansiveness short form: Validity and reliability of the MESx

**DOI:** 10.1371/journal.pone.0205373

**Published:** 2018-10-18

**Authors:** Charlie R. Crimston, Matthew J. Hornsey, Paul G. Bain, Brock Bastian

**Affiliations:** 1 School of Psychology, University of Queensland, Brisbane, Australia; 2 Department of Psychology, University of Bath, Bath, United Kingdom; 3 Melbourne School of Psychological Sciences, University of Melbourne, Melbourne, Australia; Valparaiso University, UNITED STATES

## Abstract

Moral expansiveness refers to the range of entities (human and non-human) deemed worthy of moral concern and treatment. Previous research has established that the Moral Expansiveness Scale (MES) is a powerful predictor of altruistic moral decision-making and captures a unique dimension of moral cognition. However, the length of the full MES may be restrictive for some researchers. Here we establish the reliability and validity of a reduced moral expansiveness scale, the MESx. Consistent with the full version, the MESx is strongly associated with (but not reducible to) theoretically related constructs, such as endorsement of universalism values, identification with all humanity, and connectedness to nature. The MESx also predicted measures of altruistic moral decision-making to the same degree as the full MES. Further, the MESx passed tests of discriminant validity, was unrelated to political conservatism (unlike the full MES), only mildly associated with the tendency to provide socially desirable responses, and produced moderate reliability over time. We conclude that the MESx is a psychometrically valid alternative for researchers requiring a short measure of moral expansiveness.

## Introduction

Moral expansiveness refers to the extent to which a range of entities (human and non-human) are deemed worthy of moral concern and treatment [1,2]. An individual low in moral expansiveness typically restricts concern to “close” entities; those within more traditional bounds of consideration like kin and ingroup. In contrast, a morally expansive individual extends concern beyond traditional boundaries to entities more “distant” (e.g., strangers, animals, plants).

Moral expansiveness is a way of understanding the size of a person’s moral circle. The original conceptualization of the moral circle by Singer [3] was dichotomous, characterized as the boundary distinguishing those entities deemed worthy of moral consideration from those that are not. Early psychological studies on the moral circle adopted this binary approach [4–6]. However, questions remained as to whether a dichotomous, in-or-out approach can realistically capture the multifaceted nature of moral concern [7]. In response, the Moral Expansiveness Scale (MES; [1]) was developed.

To capture the complexity of real-world moral extension, moral expansiveness is different from previous conceptualizations of the moral circle. First, moral expansiveness goes beyond a binary operationalization to reflect the graded nature of inclusion; that moral concern can span from the acknowledgement of basic moral rights through to deep and personally binding moral obligations that supersede all other considerations [2,7]. Second, rather than considering the moral inclusion of specific types of targets, moral expansiveness incorporates a broad range of human and non-human entities spanning those traditionally at the center of our moral priority to those at the periphery. Third, the moral expansiveness approach acknowledges the potential costs of moral inclusion (e.g., time and money to defend the welfare of others) [3,8].

The moral expansiveness approach was designed to examine how broadly (and intensely) individuals extend moral consideration toward a wide range of entities, allowing for novel examinations into the structure and limits of our moral worlds [2]. The original MES showed strong associations with theoretically related constructs such as a sense of oneness with humanity and nature, and endorsement of universalism values. Most importantly, it was a powerful predictor of altruistic moral decision making, such as prioritizing humanitarian concerns over personal and national self-interest, and the willingness to sacrifice one’s life to save others [1].

### The moral expansiveness scale: Standard vs. short

In the original MES [1], participants are asked to indicate the relative moral standing of 30 entities by placing each one within four moral boundaries defined as: the inner circle (representing entities worthy of the “highest level of moral concern and standing… you have a moral obligation to ensure their welfare and feel a sense of personal responsibility for their treatment”), the outer circle (“These entities deserve moderate moral concern and consideration… you are still concerned about their moral treatment, however, your sense of obligation and personal responsibility is greatly reduced”), the fringes of moral concern (“These entities deserve minimal moral concern and standing, but you are not morally obliged or personally responsible for their treatment”), and outside the moral boundary (“These entities deserve no moral concern or standing… feeling concern or personal responsibility for their moral treatment is extreme or nonsensical”). These four boundaries of morality are graded (inner circle = 3, outer circle = 2, fringes = 1, outside = 0), and an aggregate score based on the placement of the entities is calculated to reflect the expansiveness of an individual’s moral world. The original 30 MES entities spanned 10 categories (3 per category): family & friends, in-group, out-group, revered individuals, marginalized individuals, villains, high-sentience animals, low-sentience animals, plants, and environment (see Table 1 for the complete list of entities).

**Table 1:** Factor loadings across each of the 10 MES categories. Selected MESx entities with the highest loadings appear in bold.

	Factor loadings		Factor loadings
*Family & friends*		*Villains*	
**family member**	**.76**	**murderer**	**.95**
close friend	.43	terrorist	.94
partner/spouse	.39	child molester	.93
*Ingroup*		*High sentient animals*	
**citizen of your country**	**.88**	**dolphin**	**.94**
somebody from your neighborhood	.85	chimpanzee	.91
co-worker	.77	cow	.82
*Outgroup*		*Low sentient animals*	
**somebody with different religious beliefs**	**.89**	**fish**	**.96**
supporter of opposing political party	.87	chicken	.92
foreign citizen	.87	bee	.74
*Revered*		*Environment*	
**charity/aid worker**	**.86**	**old-growth forest**	**.94**
leader of your country	.79	coral reef	.87
solider of your country	.77	national park	.80
*Marginalized*		*Plants*	
**mentally challenged individual**	**.87**	**apple tree**	**.98**
LGBT+ individual	.83	rose bush	.86
refugee	.76	redwood tree	.79

However, the scale is relatively time-consuming and taxing on participants’ time and researchers’ resources. The goal of the current research is to introduce a shortened version of the MES (called MESx)–evaluating 10 entities rather than 30 –and examine if it retains its utility without sacrificing its psychometric sophistication. Over and above the advantage of a reduced completion time, a revised MES is likely to benefit from the removal of entities that are typically politically divisive. The original scale included a number of entities towards which attitudes are likely linked to political beliefs, for example refugees, terrorists, and national leaders. Therefore, a collection of entities less tied to ideological attitudes is likely to offer a cleaner measure of expansive moral concern.

Across three studies, the current research establishes the validity and reliability of the MESx. Specifically, a pilot study identified the target exemplars across the 10 MES categories to construct the shortened MESx. In Study 1, we examined the convergent and predictive validity of MESx and compared it to the full MES. In Study 2 we examined the association with social desirability, discriminant validity, and test-retest reliability of moral expansiveness for the first time. Research was approved by the School of Psychology Research Review Committee at the University of Queensland (clearance number: 14-PSYCH-PHD-63-AH).

## Pilot study: Constructing the MESx

The purpose of the pilot study was to construct the shortened scale by identifying single-entity exemplars within each of the ten original MES categories, thereby reducing the number of MES entities from 30 to 10. The chosen analytical approach used to identify the MESx items was guided by the approach taken in the construction of the full MES [1]. As the original MES categories do not represent statistically unique sub-dimensions, our goal was simply to identify a single exemplar entity from each of the pre-existing categories. In doing so, we intended to capture the broad spread of human and non-human entities that made up the full version of the MES. Given that factor loadings reflect the correlation between a variable and the underlying factor [9], responses to the complete list of 30 MES entities were gathered and these loadings were used to identity the 10 category exemplars.

## Method

One-hundred and forty-five U.S. participants (57.90% male; *M*age = 33.76, *SD* = 15.80) accepted the invitation to complete the survey online after reading a popular science article on the topic of moral decision-making. After reading the MES instructions participants completed the full MES containing all 30 of the original entities. The complete instructions are presented in S1 Appendix; additional files for those wishing to use the MESx can be found on the Open Science Framework at DOI 10.17605/OSF.IO/NC5F6.

## Results & discussion

Two participants were removed because they failed an attention check, leaving 143 for analysis. Individual factor analyses (principal axis factoring) were conducted on each of the 10 MES categories. Each category produced a single factor with generally very high entity loadings within each of the categories. Factor loadings used to identify the best individual targets are presented in Table 1. Based on these results, the ten entities selected within each category were: family member, citizen of your country, somebody with different religious beliefs, charity/aid worker, mentally challenged individual, murderer, dolphin, fish, old-growth forest, and apple tree.

Reliability analyses were conducted, with the MESx producing an alpha of .84, below that of the full MES (α = .94), but above the acceptable threshold for Cronbach’s α (0.7–0.8; [10]). Aggregate MES scores were then calculated for both the full and short versions of the scale. Unsurprisingly, the correlation between the long and short versions of the scale was also exceptionally high at *r* = .984, *p* < .001, although this value is inflated given the duplication of ten targets used in each scale. On the basis of these initial analyses, the ten exemplar targets were selected to form the MESx examined in Study 1.

## Study 1: Convergent and predictive validity of the MESx

In Study 1, our primary goal was to examine the validity of the MESx against the original scale. In assessing convergent and predictive validity, we examined responses to the MESx against key constructs and outcome measures that were used to establish the full MES [1]. Constructs used to test for convergent validity were universalism values [11], connectedness to nature [12], and identification with all humanity [13]. Constructs used to test predictive validity were two measures of altruistic moral decision-making: prosociality and willingness to self-sacrifice.

## Method

Five hundred and forty-nine U.S. participants (55.20% female; *M*age = 39.06, *SD* = 11.74) were sourced through Amazon’s Mechanical Turk. After reading the MES instructions participants were randomly assigned to either complete the full MES or the shortened MESx. Following this, all participants completed the selected measures of convergent validity, criterion measures of altruistic moral decision-making, and demographic variables.

### Convergent measures

In assessing the convergent validity of the MESx, our aim was to include measures previously used in the validation of the full scale [1]. The identification with all humanity [13] and connectedness to nature [12] scales capture a sense of oneness with humanity and with nature respectively, whereas universalism values reflect a sense of tolerance and concern for the well-being of others and for nature [11,14]. Each of these measures have been found to positively correlate with more expansive moral boundaries [1].

The connectedness to nature scale [12] uses 14 items to capture trait levels of feeling emotionally connected to the natural world. Example items in the connectedness to nature scale include: “I often feel a sense of oneness with the natural world around me”, “I think of the natural world as a community to which I belong” (1 = *strongly disagree* to 5 = *strongly agree*; α = .90). Similarly, the identification with all humanity scale [13] assesses concern toward and connection to: “the local community”, “Americans”, and “all humans everywhere”; e.g., “How close do you feel to each of the following groups”, “How much do you identify with [feel a part of, feel love toward, have concern for] each of the following?”; 1 = *not at all close* to 5 = *very close*). Responses to the 9 items assessing identification with “all humans everywhere” are summed to produce a gauge of identification with all humanity (α = .91). Finally, endorsement of universalism principles was captured using Schwartz’s [11,14] universalism values. Participants indicated the extent to which a set of 6 values represented guiding principles in their life (e.g., broadmindedness, equality; 1 = *not at all important* to 5 = *extremely important*; α = .84).

### Criterion measures

Participants completed the key measures of altruistic moral decision-making used in the original validation of the MES [1]. This included an updated version of the human and non-human prosociality measures (originally adapted from the Human Rights Choices Questionnaire; [13], and the Willingness to Self-Sacrifice measure. The prosociality measures require participants to rate the importance of competing policies that either prioritize national vs. global issues (e.g., “a—making sure America has the best hospitals in the world” vs. “b—ending slavery where it is still practiced (Sudan, etc.)”; 1 = *Item a is much more important* to *5* = *Item b is much more important*). Items were split into separate subscales where the global issues were either human (α = .82) or non-human (α = .85), each containing 5 items. The Willingness to Self-Sacrifice measure assessed participants’ hypothetical willingness to sacrifice their lives in order to save the lives of a range of human and non-human entities under threat of extinction from an evil dictator [1]. Participants were presented with 6 groups of entities (“the population of Africa”, “people in your country with an intellectual disability”, “those currently incarcerated in your country”, “chimpanzees”, “redwood trees”, and “coral reefs”) and asked to consider how many of each group would need to be killed for them to sacrifice themselves in their place (e.g., 1 = *1–10*; 2 = *10–100*; 3 = *100–1*,*000*; 4 = *10%*; 5 = *25%*; 6 = *50%*; 7 = *75%*; 8 = *90%*; 9 = *100%*; 10 = *I would never sacrifice myself*; α = .89).

### Additional measures

At the conclusion of the survey, participants responded to a range of demographic questions. In addition to age and gender, participants rated single item measures of religiosity (“How religious are you?”; 1 = *not at all religious* to 7 = *very religious*), spirituality (“How spiritual are you?”; 1 = *not at all spiritual* to 7 = *very spiritual*), nationalism (“To what extent do you feel the nation in which you live is an important part of who you are?”; 1 = *not at all important to who I am* to 7 = *extremely important to who I am*), and income (“What is your average household income?”; 1 = *less than $20*,*000* to 6 = *more than $100*,*000*). Also included were two single-item measures of political conservatism: *economic conservatism* (“Please indicate your political beliefs from left/liberal to right/conservative on issues of the economy, e.g., social welfare, government spending, tax cuts”) and *social conservatism* (“Please indicate your political beliefs from left/liberal to right/conservative on social issues, e.g., immigration, homosexual marriage, abortion”; 1 = *left/liberal* to 7 = *right/conservative*).

## Results & discussion

Thirty-one participants (5.65%) were removed because they failed an attention check, leaving 518 for analyses. Mean moral standings across both scales are presented in Table 2. The pattern of MESx largely mirrors that of the full MES, with human targets worthy of the greatest standing and concern (unless they have committed an act to lower their standing, i.e., “murderer”), followed by non-human entities and elements of the natural environment.

**Table 2:** Mean moral standing of the 10 groups and individual targets from the full and short versions (scale 0–3) of the MES. Scores are presented on the same scale for ease of comparison.

Full MES Groups	Mean (SD)	MESx Targets	Mean (SD)
1. Family & Friends	2.90 (.27)	1. Family & Friends (family member)	2.98 (.14)
2. Ingroup	2.06 (.65)	2. Marginalized (mentally challenged individual)	2.21 (.71)
3. Revered	1.90 (.67)	3. Revered (charity/aid worker)	2.08 (.71)
4. Marginalized	1.79 (.75)	4. Ingroup (American citizen)	2.05 (.68)
5. Outgroup	1.54 (.74)	5. Outgroup (different religious beliefs)	1.72 (.81)
6. Environment	1.36 (.84)	6. Animal—high sentience (dolphin)	1.48 (.92)
7. Animals—high sentience	1.30 (.84)	7. Environment (old-growth forest)	1.41 (.90)
8. Animals—low sentience	1.16 (.83)	8. Animal—low sentience (fish)	1.28 (.94)
9. Plants	1.05 (.84)	9. Plant (apple tree)	1.08 (.90)
10. Villains	0.46 (.76)	10. Villain (murderer)	0.71 (.98)

Bivariate correlations were then examined among the full and short versions of the MES and a range of demographic variables (Table 3). The full MES held no association with single-item measures of age, gender, religiosity, spirituality, nationalism and income, though we found significant negative correlations with endorsement of both economic (*r* = -.25, *p* < .001), and social political conservatism (*r* = -.18, *p* = .005). In contrast, the MESx held a weak positive correlation with age (*r* = .12, *p* = .047), but no associations with any other demographic variables, including political conservatism (likely due to the removal of politically sensitive target entities).

**Table 3:** Bivariate correlations between the MESx and demographic variables.

	Full MES	MESx
Age	.04	.12*
Gender	.05	-.03
Religiosity	-.06	-.01
Spirituality	.05	.05
Conservatism—Economy	-.25***	-.08
Conservatism—Social	-.18**	-.11
Nationalism	-.03	-.05
Income	-.02	.05

* *p* < .05.

** *p* < .01.

***p < .001

Correlations assessing the convergent and predictive validity of the MESx relative to the full MES are presented in Table 4. In line with Crimston et al. [1], the full MES produced reliable correlations with our measures of convergent validity, with greater moral expansiveness associated with stronger identification with all humanity, connectedness to nature, and endorsement of universalism values. Supporting the convergent validity of the shortened scale, the MESx also produced significant correlations with each of the convergent measures. Fisher’s comparisons of correlation strength indicated there were no significant differences between the full MES and MESx correlations on identification with all humanity (*z* = 1.36, *p* = .174), and connectedness to nature (*z* = 1.77, *p* = .077), but the full MES had a marginally stronger association with universalism (*z* = 1.93, *p* = .054).

**Table 4:** Bivariate correlations between the full MES, the MESx and measures of convergent and predictive validity.

	MES	MESx
**Convergent Validity Measures**		
Identification with all Humanity	.45***	.38***
Connectedness to Nature	.47***	.38***
Universalism	.46***	.36***
**Predictive Validity Measures**		
Prosociality—Human	.31***	.19**
Prosociality—Non-human	.34***	.30***
Willingness to Self-sacrifice	.24***	.27***

** *p* < .01.

***p < .001.

Also in line with previous findings [1], the full MES held significant and strong correlations with a willingness to prioritize human and non-human needs over ingroup needs, and a willingness to sacrifice one’s life in order to save other human and non-human entities. Similarly, the MESx produced significant positive correlations with all three measures of altruistic moral decision-making (Table 4). Crucially, there were again no significant differences between the full MES and MESx coefficients on prosociality towards other human groups (*z* = 1.38, *p* = .168), prosociality towards non-humans (*z* = .59, *p* = .555), or willingness to self-sacrifice (*z* = -.42, *p* = .675).

The findings of Study 1 lend support to the convergent and predictive validity of the MESx. As predicted—and unlike the full MES—responses on the shorter MESx were not related to political conservatism (likely due to the removal of politically sensitive target entities), and were unrelated to other demographic variables (except for a weak association with age). The MESx and the full MES also held comparably strong, significant correlations with our primary measures of convergent validity (identification with all humanity, connectedness to nature, and endorsement of universalism values). Most crucially, associations between the MESx and key criterion measures of prosociality and willingness to sacrifice were strong, and not significantly different from those held by the full MES. Overall, these findings suggest the MESx suitably captures the moral expansiveness construct with a reduced number of entities.

## Study 2: Discriminant validity and test-retest reliability of the MESx

Study 2 examined three elements of scale validation previously unexplored with regard to the MES: discriminant validity, social desirability and test-retest reliability. Convergent validity of the full MES was thoroughly explored in the original examination of the construct [1], yet discriminant validity was not investigated. Likewise, an examination of the extent to which MES responses were tied to impression management, and their stability versus variability over time, were novel lines of inquiry for the moral expansiveness construct.

## Method

Study 2 was conducted over two waves. In the first wave, participants completed the MESx and measures of discriminant validity. In the second wave (undertaken 5 weeks after the initial testing) follow-up participants completed only the MESx to examine test-retest reliability. In line with recent research [15] we estimated a follow-up response rate on Mechanical Turk of approximately 40%. Therefore, to ensure an adequately sized wave two sample (at least 200 based on sample size conventions for an estimated effect size between 0.6 and 0.8; [16]), we aimed for a sample of approximately 600 in wave one. Six hundred and nine U.S. participants (51.90% female; *M*age = 36.85, *SD* = 12.20) sourced through Amazon’s Mechanical Turk participated in the initial wave of the study. These participants completed the MESx, followed by measures of discriminant validity and social desirability.

### Discriminant validity measures

In order to assess the discriminant validity of moral expansiveness, we examined MESx responses against established psychological constructs that were predicted to be theoretically unrelated. The Rosenberg Self-Esteem Scale [17] was used to measure overall self-worth across 10 items (e.g., “On the whole, I am satisfied with myself”; 1 = *strongly disagree* to 4 = *strongly agree*; α = .93). The extraversion (e.g., “extraverted, enthusiastic”; 1 = *disagree strongly* to 7 = *agree strongly*; α = .80) and emotional stability (e.g., “calm, emotionally stable”; α = .81) dimensions of the Ten-Item Personality Inventory [18] were used to assess trait levels of these two personality dimensions. Theoretically, we could see no *a piori* reason why holding more or less expansive moral orientations should be dependent on overall self-esteem or trait levels of extraversion and emotional stability. In addition to our measures assessing discriminant validity, the 11-item version [19] of the Marlowe-Crowne Social Desirability Scale [20] was used to provide an indication of the extent to which responses on the MESx were associated with the tendency to provide overtly desirable responses (e.g., “I’m always willing to admit it when I make a mistake”; 1 = *true*, 2 = *false*; α = .78).

## Results & discussion

Twenty-five participants (4.11%) were removed because they failed attention checks, leaving 584 for analyses. In line with predictions concerning discriminant validity, individual differences in moral expansiveness as captured by the MESx were not significantly associated with trait levels of extraversion (*r* = .02, *p* = .588), emotional stability (*r* = .02, *p* = .710) or overall self-esteem (*r* = .04, *p* = .355). In addition, scores on the MESx had a significant, yet very low association with responses on the Social Desirability Scale (*r* = .09, *p* = .036).

### Test-retest reliability

Participants who completed wave one were approached 5 weeks later using the TurkPrime platform [21] to take part in the second wave. Of the initial 584 valid participants in wave one, 313 completed the second wave and were successfully tied to wave one responses (53.60%). Of the wave two participants, an additional 6 were removed because they failed an attention check, leaving 307 for the analysis. Responses to the MESx at wave one and wave two were then assessed, producing moderate test-retest reliability over a 5-week period, *r* = .61, *p* < .001.

Overall, the findings of Study 2 further support the construct validity of moral expansiveness, and specifically of the reduced MESx as a valid measure. Scores on the MESx were not associated with trait levels of extraversion, emotional stability and self-esteem, constructs for which no association was theoretically predicted. In addition, responses on the reduced moral expansiveness scale had only very mild associations with a tendency to provide socially desirable responses, suggesting that accuracy of responses in relation to self-presentation and impression management should not be a great concern for future research.

The current work assessed the test-retest reliability of moral expansiveness for the first time, with the MESx producing only moderate reliability over time. One possibility for this result may be that moral expansiveness decision-making is somewhat context dependent (for a review see [2]). Indeed, the possibility that moral expansiveness is context dependent is consistent with some earlier examinations of the binary moral circle, with previous research finding individual decisions regarding moral inclusion to be somewhat dependent on variations in context [4] and cognition [5,6]. Recent theoretical work has considered the susceptibility of our perceptions of moral standing and responsibility to various pressures and competing motivations [2,22–24]. However, additional empirical work is required on this front to provide a clearer picture of the stability of moral expansiveness decision-making, and the extent to which perceptions of moral standing and obligation are indeed flexible and context dependent.

## General discussion

The current manuscript outlines the feasibility of a reduced moral expansiveness scale, the MESx. The MESx produced high internal consistency, and responses were not tied to measures of political conservatism. Further, the MESx was strongly associated with a sense of identification with all humanity, connectedness to nature, and endorsement of universalism values, though not to the point of redundancy. Most crucially, the MESx predicted key criterion measures of altruistic moral decision-making as strongly as did the full MES. Finally, the MESx was not connected to theoretically unrelated constructs (self-esteem, extraversion, or emotional stability), was only mildly associated with the tendency to provide socially desirable responses, and produced moderate reliability over time.

Overall, the pattern of results demonstrate that the performance of the MESx is comparable to the full scale. Therefore, the MESx could be implemented as a valid measure of moral expansiveness in contexts where time or resource constraints mean the use of the full scale is unrealistic. However, the MESx should not be viewed as a direct replacement for the original scale. Even though the reduced association with political orientation means the MESx might provide a cleaner measure of moral expansiveness, the complete scale is arguably better suited to fully capture the nuanced and multifaceted nature of moral boundary decision-making. Rather, the MESx should be viewed as part of a suite of tools for examining moral expansiveness: the shortened scale for resource-constrained researchers, the complete scale for those who are focused on observing nuances within the moral circle, and the adapted version designed to assess morally expansive decision-making throughout development [25].

In conclusion, the current research establishes the MESx as a psychometrically valid measure of attributions of moral standing and perceived responsibility. Although it is not able to capture as detailed an assessment of moral expansiveness as the full version, the reduced scale still incorporates all of the central elements of the underlying construct—a graded approach of moral concern, broad range of entities, and the consideration of personal costs—without sacrificing predictive power or psychometric sophistication. As such, the MESx is a valid and reliable gauge of moral expansiveness suitable for resource-constrained researchers and/or time-constrained participants.

## Supporting information

S1 Appendix MESx instructions. (DOCX)
