# Supplementary material for: Moral expansiveness short form: Validity and reliability of the MESx
Source: PLoS One. 2018 Oct 18;13(10):e0205373. doi: 10.1371/journal.pone.0205373 (PMC6193647; doi:10.1371/journal.pone.0205373)

**Supporting Information**

**S1 Appendix. MESx Instructions**

**Moral Circles**

People sometimes talk about **'circles of moral concern'**. These circles are simple ways to make sense of the levels of moral consideration we have for different entities (e.g., people, animals, and the environment).
 
**Where we place these entities within our moral circles is important as it reflects their moral worth, and has direct consequences for how we treat them.**

On the following page you are given the opportunity to organise a range of entities and place them within your own moral circles that reflect your individual views and feelings.


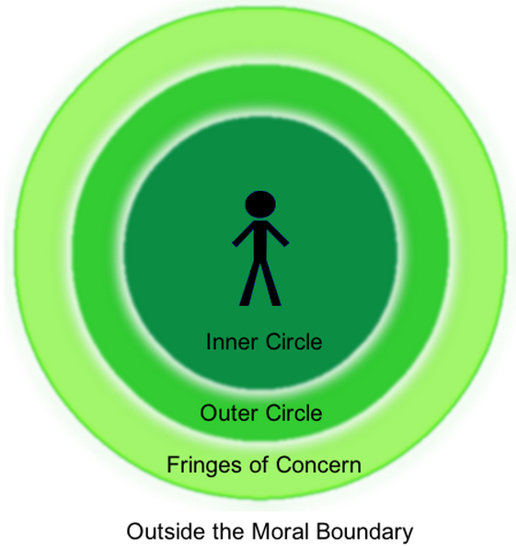


**Please read the four boundary descriptions below carefully before completing the moral circle task.**


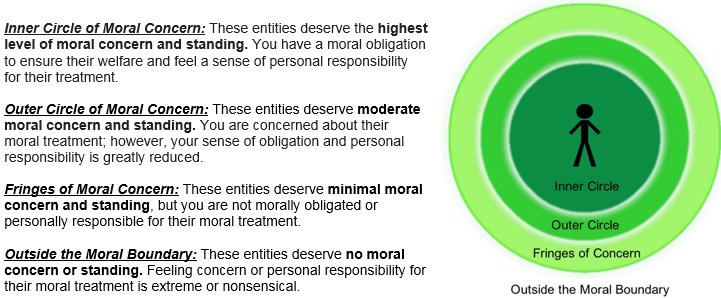


*Having carefully read these descriptions*, please consider the **level of moral concern you personally have for each of the entities below** and drop each one into the appropriate moral circle box on the right. 

Please note, there are no right or wrong answers - we just want to know your opinion.


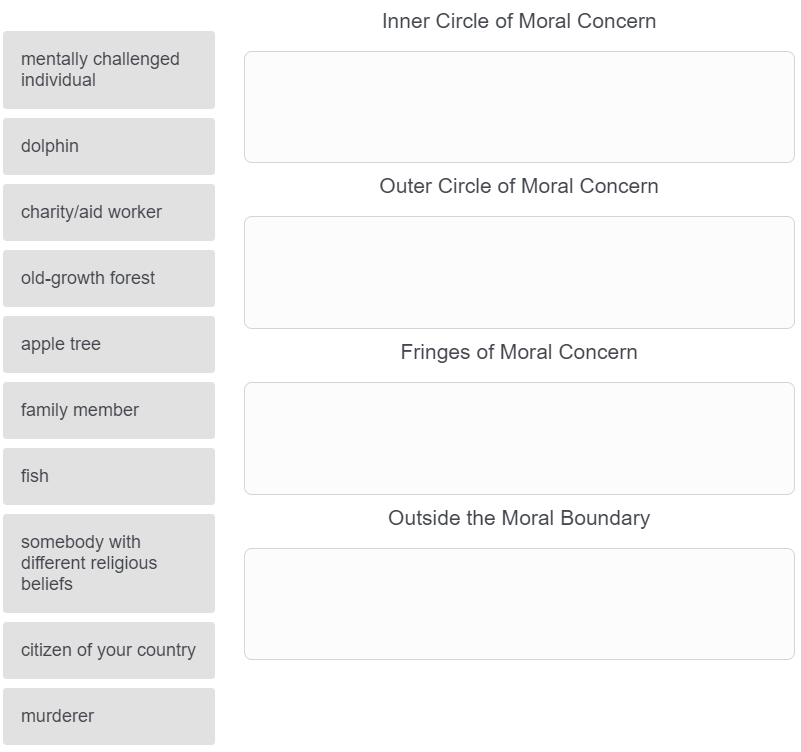

Supplement: S1 Appendix — (DOCX) [file pone.0205373.s001.docx]
